# Clinician–patient communication about emergency aerial medical evacuation in case of infectious disease

**DOI:** 10.1093/jtm/taad014

**Published:** 2023-01-31

**Authors:** Charlotte Albury, Madeleine Tremblett, Helena Webb, Rachna Begh, Rebecca Barnes, Wendy Lawrence, Nichola Walmsley, Deborah Groenewald, Marise Caunter, Dipti Patel

**Affiliations:** Nuffield Department of Primary Care Health Sciences, University of Oxford, Oxford, UK; Nuffield Department of Primary Care Health Sciences, University of Oxford, Oxford, UK; University of Nottingham, Nottingham, UK; Nuffield Department of Primary Care Health Sciences, University of Oxford, Oxford, UK; Nuffield Department of Primary Care Health Sciences, University of Oxford, Oxford, UK; Healix Group, Surrey, UK; Healix Group, Surrey, UK; Healix Group, Surrey, UK; Rural Planning Services, Occupational Health, UK; UK Foreign Commonwealth and Development Office, London, UK

**Keywords:** Air ambulances, emergency evacuation, clinical communication, patient provider interaction, aerial medical evacuation, infection, patient transport

## Abstract

Conversation analysis of clinician–patient telephone consultations showed that communicating the process and possibility of aerial medical evacuation with people who may require it in future could support better understanding of the process and informed decision-making prior to travel. We identified clear steps clinicians can take to do this.

Aerial medical evacuation (AME) refers to the removal of patients from one site to a medical facility elsewhere using medically equipped air ambulances.^1^ In cases of certain infectious diseases it may be necessary to isolate patients in a patient isolation unit (PIU) or ‘pod’ during AME to reduce the risk of transmission of infection to others. Used to transfer patients within and between countries during outbreaks of infectious disease,^2^ during the COVID-19 pandemic AME became an area of ongoing need.^3^ With projections indicating current increases in pandemics will likely continue,^4^ the need for AME will do the same. However, AME in case of infectious disease is an under researched area. There is limited information about the processes and procedures of AME in case of infectious disease,^5^ and no research or guidance on how to communicate these to patients. A 2019 systematic review aimed to evaluate the processes and procedures used, including pre-flight, in-flight and post-flight.^6^ The review highlighted the importance of effective communication, but identified a dearth of studies in this area, with just one study detailing communication with patients during the flight, and none examining pre-flight communication.

There are specific barriers to clear and effective communication with patients during AME, including patient sedation or illness, patient anxiety, difficulties because of PIU structure, healthcare worker personal protective equipment and background aircraft noise. With limitations to effective two-way communication ‘during’ AME, patients may face an unfamiliar and distressing situation without opportunity to fully understand what is happening, and plans for what will happen next. Taking the opportunity to discuss the process with individuals with increased risk of needing AME for a serious communicable disease ‘in future’ could ensure they are better prepared, able to make informed decisions about international travel, understand the risks in accessing care when travelling to areas where AME may be necessary and what processes may occur.

**Figure 1:**
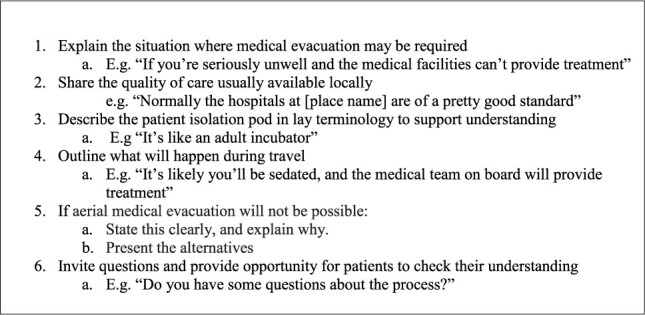
Communicating emergency AME contingency plans with patients in case of infectious disease

**Table 1:** Transcribed excerpts from calls between clinicians and British Government employees, or their dependents, who were about to travel internationally

	Example number	Jeffersonian transcription^a^	Verbatim transcription
Opportunity for questions about AME and repatriation procedure	Example 1	If things did (0.2) go: really badly and i- in the very unlikely event that I got very ↓i:ll (0.2) then is there some kind of plan ↓bee:,	If things did go really badly and in the very unlikely event that I got very ill then is there some kind of plan B?
	Example 2	So is it eh- I mean > to be honest< I don’t know much about thi:s, so is it normally the case that you you would like (.) put somebody in some kind of capsule to isolate them and bring them back. (0.9) Is that some standard procedure?	So is it, I mean, to be honest, I don’t know much about this. So is it normally the case that you would put somebody in some kind of capsule to isolate them and bring them back. Is that some standard procedure?”
Clarity on the details of PIUs	Example 3	Thank you >for telling me<. (.) Uh::m (1.0) I:: could I ask a first question wha-what are these like air pods (0.2) li::ke?	Thank you for telling me. Could I ask a first question? What are these air pods like?
	Example 4	Patient: Yeah, so w- ↑how does that work then. (.) Do they- do they put you in the pod and then (0.5) you’ve got like, (0.7) y- (1.0) you-you can’t, (0.7) yeah so have you got a- has someone gotta push you alo::ng ↓inside this like ↓isolation cube and (0.5) load you onto a pla::ne (0.7) in the-Clinician: i-i-Exactly.	Patient: Yeah, how does that work then? Do they put you in the pod and then… has someone got to push you along inside this like isolation cube and load you onto a plane?Clinician: Exactly.
Previously unknown information about the possibility and processes of AME	Example 5	Yeah, (.) thank you I appreciate you saying cos it is (0.3) it’s good to know so you’re not finding this stuff out when it happens a::nd (.) it could be a bit worrying, (.) so thank you for giving me the heads up, (.) that’s good to know.	Yeah, thank you I appreciate you saying because it is good to know so you’re not finding this stuff out when it happens, and it could be a bit worrying, so thank you for giving me the heads up. That’s good to know.

^a^Jeffersonian transcription is an established convention that records how talk is delivered as well as what is said, supporting rigorous CA^^7^^. A simplified version is presented here, alongside verbatim equivalents. We use the following notation: ↓ or ↑—notable and/or sharp rises or falls in pitch; ?—rising tone; , —gently rising tone; :—elongation of the immediately prior sound, where the number of colons shows the length of elongation; ><—the enclosed talk was hearably faster than the surrounding talk; (.)–a pause of <0.3 s; (0.3)—numbers in parentheses indicate pauses in talk, measured in tenths of a second.

We aimed to identify how clinicians communicate with people about AME prior to international travel, and to highlight recommendations for practice. To address our aim, we analysed 20 recorded telephone calls between clinicians and British Government employees who were about to travel internationally. These calls were made during the height of the COVID-19 pandemic (September 2020 to July 2021). The aims of the calls were to discuss: (i) COVID-19 risk, (ii) the possibility of AME and repatriation to the UK, outlining a contingency plan should a person become seriously unwell and need AME and (iii) the process of AME.

Clinicians providing healthcare assistance to British Government employees and their families when abroad offer a range of services including pre-travel risk assessment and advice, on-going medical case management and emergency medical evacuations. Calls are recorded routinely. We sought post-hoc consent for analysis from clinicians and relevant patients (ethical approval: CUREC R75138/RE001). Clinicians carrying out calls were nurses with experience in travel medicine, emergency medicine, general practice or intensive care. Government staff were mostly based in UK diplomatic missions.

We used conversation analysis^8^ (CA) to analyse these data. CA is a well-established method for studying communication and social interaction. It focuses on the sequential organisation of interaction, examining how each conversational turn gives rise to, and creates the context for, the next. CA takes a systematic approach to examining what actions are achieved with talk, and enables creation of an evidence base of effective practice. This in-depth approach is commonly used to study clinical communication, including shedding light on interactions in emergency medical settings,^9^ and about illness progression,^10^ and to make recommendations for practice.

Albury led analysis, with input from Tremblett, Webb, and Begh who reviewed ongoing findings and suggested additional analytic foci.

Our analysis highlighted six steps clinicians can use to communicate AME contingency plans (Figure 1). We found that taking opportunity to do this, and discuss the possibility and processes of AME prior to travel played an important role providing:

1. An opportunity for questions about AME and repatriation procedures

Discussing AME provided opportunity for patients to ask for more detail on specific aspects of the process, including if there is a plan in place should they become seriously ill (Table 1, Example 1), or to find out more about AME procedures (Table 1, Example 2).

1. Clarity on the details of PIUs

Many patients showed that they were unfamiliar with PIUs and used these conversations as opportunity to seek clarity on what they are ‘like’ (Table 1, Example 3), and check their understanding (Table 1, Example 4).

1. Previously unknown information about the possibility and processes of AME

After discussing AME with a clinician, patients displayed that receiving this information was helpful and useful. For example, the patient in Table 1, Example 5, states that it would be worrying to find out information about AME at the time, and its ‘good to know’ in advance.

Following these discussions, individuals were in the position to make a more informed decision about travel, with the knowledge of the risks and contingencies in place.

A strength was our focus on real calls from a unique data set of calls focusing on AME contingency planning during COVID-19, which were not subject to recall bias. Whilst an in-depth CA can be conducted with 20 recordings, a limitation is that these calls were conducted by a small team, comprising three consenting clinicians. Subsequent studies might seek greater clinician variation.

In conclusion, we found that following the recommendations in Figure 1 to communicate the process and possibility of AME with those who may require it in future could support better informed decision-making prior to travel. People showed that many aspects of the process were unfamiliar, including the concept of PIUs, and these conversations presented an opportunity to find out about the process of AME, ask questions and have PIUs explained in ‘lay language’, which people indicated was helpful in supporting understanding.

We recommend clinicians communicate AME plans in advance with those with a higher likelihood of requiring AME for a serious communicable disease (for example, those travelling during a pandemic, or working on an outbreak response). This can support informed decision-making about travel, and clearer understanding of AME processes for those who may need them.

## Funding

The UK Foreign Commonwealth and Development Office.

## Conflict of interest

In 2022, C.A. was contracted qualitative methodologist for the Behavioural Insights Team (BIT) for which she was paid personally. She has worked as a consultant qualitative methodologist for Wildfowl Wetlands Trust, Linney Create and Adelphi Real World, and received personal payment. D.P. is chief medical officer for the UK Foreign Commonwealth and Development Office. W.L., D.G. and N.W. work with the Healix Group, whose team of clinicians delivered the conversations we sought to analyse. The other authors declare no conflicts.

## Data availability

The data underlying this article cannot be shared publicly to protect the privacy of individuals that participated in the study.
